# Transgenerational Developmental Effects of Immune Priming in the Red Flour Beetle *Tribolium castaneum*

**DOI:** 10.3389/fphys.2019.00098

**Published:** 2019-02-19

**Authors:** Nora K.E. Schulz, Marie Pauline Sell, Kevin Ferro, Nico Kleinhölting, Joachim Kurtz

**Affiliations:** Institute for Evolution and Biodiversity, University of Münster, Münster, Germany

**Keywords:** innate immunity, immune priming, transgenerational effects, *Tribolium castaneum*, *Bacillus thuringiensis*, host parasite co-evolution, bacterial infection, oral infection

## Abstract

Immune priming, the increased chance to survive a secondary encounter with a pathogen, has been described for many invertebrate species, which lack the classical adaptive immune system of vertebrates. Priming can be specific even for closely related bacterial strains, last up to the entire lifespan of an individual, and in some species, it can also be transferred to the offspring and is then called transgenerational immune priming (TGIP). In the red flour beetle *Tribolium castaneum*, a pest of stored grains, TGIP has even been shown to be transferred paternally after injection of adult beetles with heat-killed *Bacillus thuringiensis*. Here we studied whether TGIP in *T. castaneum* is also transferred to the second filial generation, whether it can also occur after oral and injection priming of larvae and whether it has effects on offspring development. We found that paternal priming with *B. thuringiensis* does not only protect the first but also the second offspring generation. Also, fitness costs of the immune priming became apparent, when the first filial generation produced fewer offspring. Furthermore, we used two different routes of exposure to prime larvae, either by injecting them with heat-killed bacteria or orally feeding them *B. thuringiensis* spore culture supernatant. Neither of the parental larval priming methods led to any direct benefits regarding offspring resistance. However, the injections slowed down development of the injected individuals, while oral priming with both a pathogenic and a non-pathogenic strain of *B. thuringiensis* delayed offspring development. The long-lasting transgenerational nature of immune priming and its impact on offspring development indicate that potentially underlying epigenetic modifications might be stable over several generations. Therefore, this form of phenotypic plasticity might impact pest control and should be considered when using products of bacterial origin against insects.

## Introduction

Over the last decade a wealth of new evidence has been put forward to demonstrate that invertebrate immune systems can possess forms of immune memory and are sometimes capable of highly specific responses (Contreras-Garduño et al., 2016; Milutinović and Kurtz, 2016; Cooper and Eleftherianos, 2017). The phenomenon enabling a stronger and faster immune response upon secondary infection has been termed immune priming and shows parallels in memory and specificity to trained immunity of vertebrates (Little and Kraaijeveld, 2004; Kurtz, 2005; Netea et al., 2011; Kurtz and Armitage, 2017; Melillo et al., 2018). The trigger, specificity and duration of the priming can be extremely diverse. Immune priming can be achieved by introducing a sublethal dose of the parasite, an incapacitated, e.g., heat killed agent or using only specific molecules from the original pathogen, e.g., lipopolysaccharides (Contreras-Garduño et al., 2016; Milutinović and Kurtz, 2016). Also, the route how the elicitor is introduced can vary, similar to differences in the route of infection in nature. For experiments involving priming, the priming agent is most commonly introduced via septic wounding and deposition into the haemocoel or orally via feeding (Milutinović and Kurtz, 2016). Furthermore, also abiotic factors, e.g., thermal exposure have been shown to prompt this phenomenon (Wojda and Taszłow, 2013; Eggert et al., 2015).

Additionally, the duration of immune priming effects differs dramatically. In some cases, protection lasts across different life stages, and throughout the entire life span of an individual (Pham et al., 2007; Thomas and Rudolf, 2010; Khan et al., 2016). In some cases, the immune priming is even transferred to the offspring generation (Milutinović and Kurtz, 2016; Dhinaut et al., 2018; Roth et al., 2018). This transgenerational immune priming (TGIP) can occur through either parent. While for the maternal side, the direct transfer of bacterial particles bound to egg-yolk protein vitellogenin has been shown to be involved in certain systems (Salmela et al., 2015), the detailed mechanistic underpinnings of immune priming in general and paternal TGIP in particular still remain to be discovered (Milutinović et al., 2016).

As with any other immune response also the fitness costs of immune priming including those for storing the information have to be considered. These costs are not constraint to a direct reduction in fertility but can also become visible in delayed development or smaller body mass if the priming occurs before the organism reaches maturity. Furthermore, negative effects might only become visible in the offspring generation. In the Coleopteran, *Tenebrio molitor*, maternal priming prolonged offspring larval development (Zanchi et al., 2011) and the strength of this effect depended on the Gram type of the bacteria used for priming (Dhinaut et al., 2018). Immune priming beneficial to the mother can even increase offspring susceptibility to the same parasite (Vantaux et al., 2014). These are all factors demonstrating the complexity of immune priming and showing that this term probably covers several distinct phenomena (Pradeu and Du Pasquier, 2018). It makes predicting host-parasite co-evolution and the emergence of resistance against bacterial pesticides much more difficult if we consider that several forms of immune priming can occur in the same species across different life stages and generations with different consequences.

Immune priming has been studied intensively in the red flour beetle *Tribolium castaneum*, which is a widely abundant pest of stored grains. In this beetle, immune priming has been demonstrated in different life stages, i.e., larvae and adults, as well as within and across generations (Milutinović et al., 2016). In this species, TGIP can occur via both parents (Roth et al., 2010). Previously, mainly two different routes of priming and infection have been used with the beetle. Oral infections with spores only work in larvae and the protective benefits of priming with the supernatant of the spore culture have so far only been studied within generation, mostly even within life stage (Milutinović et al., 2014; Futo et al., 2017; Greenwood et al., 2017). Therefore, the effectiveness of the priming was only confirmed for a few days after exposure. The other priming and infection method uses vegetative cells, which are heat-killed for the priming and are directly introduced into the body cavity via septic wounding (Khan et al., 2016; Milutinović et al., 2016; Tate et al., 2017). In this case, immune priming of adults can be transferred to their offspring and a protection against infection can still be observed in the adults of the offspring generation (Roth et al., 2010; Eggert et al., 2014). But, these different priming techniques and routes of infection lead to different responses as is evident in differential gene expression and immune system activity (Behrens et al., 2014). The pathogen used in most studies of priming in *T. castaneum* is the entomopathogenic and endospore forming bacterium *Bacillus thuringiensis* (Jurat-Fuentes and Jackson, 2012). Proteins from *B. thuringiensis*, so-called Cry toxins are widely used for their insecticidal activity in transgenic crops (Pardo-López et al., 2013; Lacey et al., 2015). Therefore, the study of immune priming in this host parasite model system does not only advance basic research and our understanding of the invertebrate immune system but is also helpful for applied approaches and improving insect control strategies.

With our study we shed further light on the different forms of immune priming against *B. thuringiensis* that can be observed in *T. castaneum*. We here investigated the transgenerational effects caused by three different types of priming, i.e., priming by injection of larvae and male adults and oral priming of larvae by monitoring the development, fitness and survival of bacterial infection (challenge). As paternal TGIP so far has only been tested in the first offspring generation (Roth et al., 2010), we here expanded the experimental time frame to include the adult F_2_ generation, investigating whether TGIP is a multigenerational phenomenon extended to more than one subsequent generation. Studies on larval priming have been mainly focused on within generation immune benefits (Milutinović et al., 2016). We therefore here wanted to investigate whether larval TGIP via the oral or septic wounding infection route exists and whether the offspring is affected in a different way by parental treatment.

## Materials and Methods

### Model Organisms

Beetles were derived from a population originally collected in the wild in Croatia in 2010 (Milutinović et al., 2013). Until the start of the experiment, beetles were kept in populations of more than 2,000 individuals in plastic boxes with heat sterilized (75°C) organic wheat flour (type 550) enriched with 5% brewer’s yeast. Standard breeding conditions were set at 70% humidity and 30°C with a 12 h light/dark cycle.

In all priming treatments and infections, different entomopathogenic gram-positive *B. thuringiensis* strains were used. *B. thuringiensis* and its Cry toxins are widely used as insecticides and together with *T. castaneum* form a well-established system to study host parasite co-evolution (Roth et al., 2009; Contreras et al., 2013; Milutinović et al., 2013; Pardo-López et al., 2013). For the different priming methods, we used the *B. thuringiensis* strains, which proved most effective in previous studies (Roth et al., 2010; Milutinović et al., 2014). For priming and challenge by injection we used vegetative cells from *B. thuringiensis* (*Bt*) strain DSM 2046 (German Collection of Microorganisms and Cell Cultures, DSMZ). For the treatments concerning priming and infection by oral uptake, spores and supernatant from *Bt morrisoni bv. tenebrionis* spore cultures *(Btt*, Bacillus Genetic Stock Center, Ohio State University, Ohio, United States) were used. Additionally, *Bt407cry^-^* (*Bt407*, kindly provided by Dr. Christina Nielsen-Leroux, Institute National de la Recherche Agronomique, La Minière, 78285 Guyancourt Cedex, France) served as a negative control in the oral priming experiment, as it does not produce Cry toxins and does not lead to immune priming nor mortality upon ingestion (Milutinović et al., 2013, 2014).

### Paternal Transgenerational Immune Priming of Adults

**FIGURE 1:**
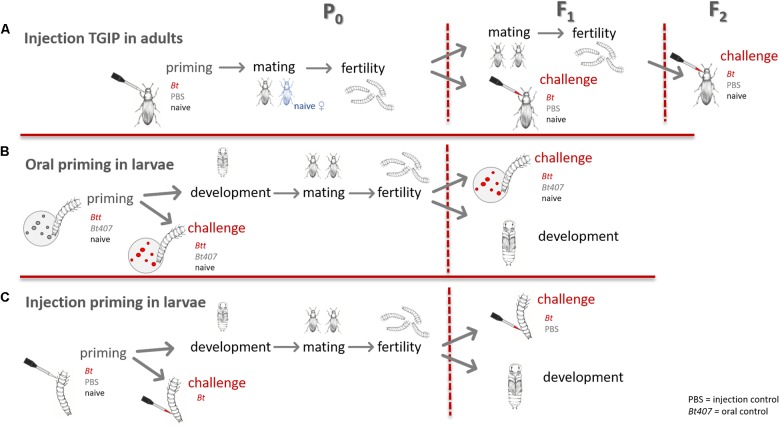
Overview of the experimental design. **(A)** TGIP by injection in adults. **(B)** Oral immune priming of larvae. **(C)** Injection immune priming of larvae.

In this experiment we wanted to investigate, whether paternal TGIP persists past the first filial (F_1_) generation (Roth et al., 2010; Eggert et al., 2014) and therefore provides survival advantages upon *Bt* infection to the second filial (F_2_) generation. Additionally, we measured the fertility of the primed males and their offspring to determine potential costs of TGIP. For an overview of the experimental design see Figure 1.

#### Injection Priming of the Parental (P_0_) Generation

To set up the P_0_ generation for this experiment around 2000 beetles from a general stock population were put into a plastic box containing 250 g of flour with yeast. After an oviposition period of 24 h the adults were sieved off and put into a new box for a second 24 h oviposition period. When the offspring had reached the pupal stage, their sex was determined, and all beetles were kept individually from here on onward.

For the priming injections one week after eclosion, 60 male adults were either injected with heat-killed *Bt* suspended in PBS at a concentration of 1^∗^10^9^ cells per ml (injection priming), PBS only to control for the wounding (priming control) or left naïve. The priming suspension was directly injected into the dorsal vessel by dorsally puncturing the epidermis between head and pronotum in a flat angle to minimize tissue damage. Heat-killed *Bt* were produced from an overnight culture as previously described (Roth et al., 2009; Ferro et al., 2017). A nanoinjector (Drummond Nanoject II) was used for this procedure with the injection volume set to 18.4 nl (∼20,000 cells per injection in the *Bt* treatment). Survival after the priming procedure was recorded 24 h later.

#### Mating and Fitness of P_0_ and F_1_ Generation

Single mating pairs with naïve, virgin females were set up (*n* = 39–57). Mating pairs were kept in plastic vials containing 6 g of flour and left to lay eggs for two consecutive 3-day long oviposition periods. Thirteen days after the end of the respective oviposition period, larvae were counted for each pair and individualized into 96 well plates with flour. For the analysis, data from both oviposition periods were combined.

The sex of the offspring was determined when they had reached the pupal stage. One female and one male offspring from each single pair formed a new mating pair to produce the F_2_ generation, leading to mating of full siblings (*n* = 29–53). Mating, oviposition and individualization of offspring larvae were carried out in the same way as described for the parental generation with the exception of the oviposition periods being shortened to 24 h. The fertility of F_1_ pairs was recorded as live larvae 12 days post oviposition (dpo).

#### Bacterial Injection Challenge of Adults of F_1_ and F_2_ Generation

The priming of adult males of *T. castaneum* with heat-killed *Bt* leads to an increased survival rate in their adult offspring when infected with a potentially lethal dose of the same bacteria (Eggert et al., 2014). Whether this phenomenon is also transferred to subsequent generations has so far not been investigated. We therefore exposed individuals of the F_1_ and F_2_ generation to a bacterial challenge after the P_0_ generation had received a priming treatment. Bacteria were cultured, washed and their concentration in PBS adjusted as for the priming procedure without the heat-killing step (2.2.1). One week after eclosion animals of both sexes were injected with a volume of 18.4 nl. The injection either contained *Bt* cells at a concentration 10^7^ vegetative bacterial cells per ml (∼200 cells per injection) in PBS (injection challenge) or only PBS as a control (injection control) and was performed in the same manner as described for priming (2.2.1). A second control consisted of a naïve group that received no injections. In the F_1_ generation, three adult siblings from each family were used, one for each challenge treatment (*n* = 31–44). This was the same for the F_2_ generation, but here the challenge was performed on adults originating from two consecutive ovipositions of the same families (oviposition 1: *n* = 16–42, oviposition 2: *n* = 24–45). Injections were carried out in the same manner as for the priming treatment (2.2.1). Afterward, the beetles were kept in individual glass vials and their survival was recorded 24 h post challenge.

### Transgenerational Effects of Immune Priming in Larvae

Within generation immune priming of *T. castaneum* larvae with *B. thuringiensis* can be achieved by two different exposure routes: first, septic priming can be achieved by the introduction of heat-killed vegetative cells into the hemolymph, which can be done by pricking the cuticle with a needle that was dipped into a suspension of heat-killed bacteria or by injection of heat-killed bacteria in the body cavity. Second, oral priming can be achieved by oral ingestion of spore culture supernatant (Behrens et al., 2014; Ferro et al., 2017). For this, the supernatant derived from a centrifuged *B. thuringiensis* spore culture is sterile filtered (0.2 μm) and then used for the preparation of the priming diet (Milutinović et al., 2014). It is so far unknown, which bacteria-derived components remain in the filtered supernatant and might elicit the immune priming. Here, we investigated the costs and transgenerational effects of the two different larval priming methods.

#### Oral Immune Priming of Larvae

For the culturing and sporulation of *B. thuringiensis tenebrionis* we followed the method given in Milutinović et al. (2013). Milutinović et al. (2014) describe the methodology to orally prime larvae with *Btt* spore supernatant. In short, for the oral priming the spore supernatant is provided to the beetle by mixing with flour and PBS, pipetting the mixture into a 96 well plate and letting the diet dry to form flour disks. In addition to the *Btt* treatment (oral priming), *Bt407* was used as a negative control (priming control) because the supernatant from its spore culture does not provide a priming effect (Milutinović et al., 2014). As a third group a naive control was included with pure PBS to produce the flour disks (naïve).

The P_0_ generation originated from approximately 1000 beetles from our stock population ovipositing for 24 h. Larvae of the P_0_ generation were exposed to the priming diets 14 dpo for 24 h (*n* = 320). After the priming, a subgroup of the primed larvae was transferred onto naïve flour disks, on which they remained until the oral challenge or were used in producing the F_1_ generation.

#### Oral Immune Challenge of Larvae

The within generation challenge was performed to confirm successful priming. The challenge took place 19 dpo, i.e., 5 days after the exposure to the priming diet, in a full factorial design. Besides the challenge diet of *Btt* spores (oral challenge), two controls were included using either *Bt407* spores, which are not infective to the beetles (challenge control) or flour disks prepared with pure PBS (naïve) (*n* = 40). The same bacteria culturing sporulation assay as for the oral priming was used (Milutinović et al., 2014). The spore concentration was adjusted to 5^∗^10^9^ spores per mL. Larvae stayed on their respective flour disks for the rest of the experiment. Survival after challenge was recorded daily for the next 8 days.

#### Costs of Oral Immune Priming in Larvae

To identify potential costs of the oral immune priming, we monitored the development of the larvae until adulthood for the three priming treatment groups (oral priming, priming control, and naïve). In the P_0_ generation, pupation rates were checked 23 dpo and the proportion of eclosed adults was recorded 27 dpo. In a subgroup of treated larvae, the sex of the individuals was determined during pupal stage and once they had reached sexual maturity (5 days post eclosion) single mating pairs were formed within each priming treatment (*n* = 57–66). Pairs were allowed to mate and produce eggs for two consecutive 24 h oviposition periods. Afterward, the adults were sieved off and offspring larvae were counted 14 dpo to estimate fertility. For the analysis, data from both oviposition periods were combined. To determine whether the oral immune priming produced any costs, which only become visible in the F_1_ generation, the development of a subgroup of this larvae was monitored. The offspring larvae were individualized 14 dpo and kept in lose flour the entire time. They were checked for pupation between 19 and 23 dpo and their eclosion rates were noted 28 dpo.

#### Oral Immune Challenge of F_1_ Generation Larvae

Furthermore, we wanted to know whether the oral immune priming of larvae can also be transferred to the F_1_ generation, as has been observed in the priming of adult *T. castaneum* (Roth et al., 2010; Tate et al., 2017). To answer this, a subgroup of the F_1_ generation was orally challenged as well. This group was produced by the mating of single pairs, with individuals coming from the same priming group. One individual from each mating pair was used for each of the three challenge treatments (*n* = 71–76). The challenge was conducted in a similar manner as for the P_0_ generation, but without the naïve control. Instead it included two different spore concentrations to counteract the possibility of too high or too low mortality rates. The spore concentration was set to either 1^∗^10^10^ spores per ml (high dose) or 5^∗^10^9^ spores per ml (low dose). Larvae were put on naïve flour disks at 14 dpo to ensure similar development as in the P_0_ generation and to avoid early pupation, as the development in lose flour is considerably faster than on flour disks. The challenge took place 19 dpo and again survival was monitored for 8 days.

#### Injection Immune Priming of Larvae

Priming by injection with heat killed *Bt* cells (injection priming) took place 14 dpo. The larvae for this experiment came from a 24 h oviposition of ∼1000 beetles from our stock population. The procedure also included an injection control in which only PBS was used and a naïve group (*n* = 244). Heat-killed priming bacteria were produced as described above (2.2.1). Priming injections had a volume of 18.4 nl and were placed in a flat-angle laterally under the epidermis of the third-last segment using a nanoinjector (Drummond Nanoject II). The bacterial concentration was adjusted to 1^∗^10^9^ cells per ml (∼20,000 cells per larvae). After the injection, larvae were kept individually in 96 well plates containing flour.

#### Injection Immune Challenge of Larvae

We performed a within life stage injection challenge to confirm the success of the priming. During the bacterial challenge 19 dpo, i.e., 5 days post priming a subgroup of the animals was injected with 18.4 nl of either vegetative *Bt* cells at a concentration of 1^∗^10^7^ cells per ml suspended in PBS (injection challenge) or only PBS (injection control) (*n* = 48). Challenge injections were placed in the dorsal vessel at a flat angle dorsally under the epidermis of the first thoracic segment to minimize tissue damage. After the challenge injection, larvae were continued to be kept individually, and their survival was checked 7 days later.

#### Costs of Injection Immune Priming in Larvae

Also, for the injection priming of larvae, we wanted to test whether the treatment was costly and impacted the development. We therefore checked the proportion of pupae in a subgroup of the P_0_ generation (*n* = 196) 23 dpo and the proportion of eclosed adults in the F_1_ generation (*n* = 72–103) 27 dpo. The F_1_ generation was produced from single mating pairs within a priming treatment and offspring larvae were individualized 14 dpo, i.e., the age their parents had been primed.

#### Injection Immune Challenge of F_1_ Generation Larvae

Injection challenge was performed on a subgroup of the F_1_ generation larvae, to discover whether a priming benefit and increased protection is transferred to the offspring. The F_1_ generation was produced from single mating pairs within the same priming group, which produced eggs for two consecutive 24 h periods (*n* = 96). The challenge procedure was the same as in the P_0_ generation. Larvae were injected 19 dpo with 18.4 nl of either vegetative *Bt* cells at a concentration of 1^∗^10^7^ cells per ml suspended in PBS or only PBS. Again, survival was measured after 7 days.

### Statistical Analysis

All statistical analyses were performed in R (R Development Core Team, 2008) using RStudio (R Studio Team, 2015). Additional packages utilized included: MASS (Venables and Ripley, 2002), lme4 (Bates et al., 2015), multcomp (Hothorn et al., 2008), and survival (Therneau and Grambsch, 2000). Data concerning larval survival and development until pupation were tested in a Cox proportional hazard analysis, after it had been ensured that the assumption of hazards being proportional over time had been fulfilled. When this was not the case, generalized linear mixed effects models (GLMM) with a binomial distribution and experimental block as random factor were applied on data for one specific time point for pupation rates. This method was also used to examine eclosion rate. Tukey honest difference (THD) was applied *post hoc* to determine significant differences between individual treatment groups, while adjusting the *p*-values for multiple testing. *X*^2^-tests were used to analyze survival after injection challenge in cases for which random factors did not apply.

## Results

### TGIP by Injection of Adults Is Transmitted to the F_2_ Generation

**FIGURE 2:**
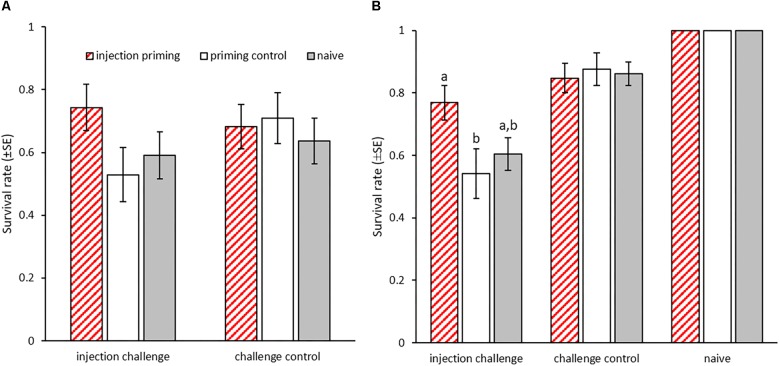
Survival of bacterial injection challenge after paternal TGIP. Male adults were primed by injection with heat-killed *B. thuringiensis*. **(A)** Survival rates 24 h after injection challenge with live *B. thuringiensis* of the adult F_1_ generation according to paternal priming (*n* = 31–44). **(B)** Survival rates 24 h after injection challenge with live *B. thuringiensis* of the adult F2 generation according to grand-paternal priming (two experimental blocks: *n* = 16–42 and *n* = 24–45). Different letters indicate significant differences at *p* < 0.05.

In *T. castaneum*, immune priming by injection of heat-killed bacteria into adults has been shown to provide a survival benefit upon bacterial challenge to their offspring (i.e., F_1_ generation) when they had become adults themselves (Roth et al., 2010). This effect was observed for both mothers and fathers. Focusing on the paternal priming route, we here investigated whether such trans-generational immune priming (TGIP) is also transferred further, to the F_2_ generation. Before challenging the F_2_ generation, we first wanted to confirm successful TGIP in the adults of the F_1_ generation. However, in the F_1_ generation, we observed an unusually high death rate in the control beetles that were injected with buffer only (i.e., challenge control) instead of the bacterial challenge (i.e., injection challenge). In consequence, we did not observe any significant differences in mortality between those beetles, regardless of paternal priming (*N* = 232, *X*^2^ = 0.707, *p* = 0.4; Figure 2A). However, within the beetles that received an injection challenge, there was a tendency toward TGIP, as we observed a trend toward increased survival in the paternally injection primed group compared to the priming control (*N* = 69, *X^2^* = 3.401, *p* = 0.065; Figure 2A). As expected, there was no such difference between the priming treatments in the challenge control (*N* = 119, *X*^2^ = 0.473, *p* = 0.78; Figure 2A).

We then tested whether TGIP is also passed on to the successive generation. The challenge of the adult F_2_ generation proved to be effective, as significantly more beetles died after injection with live bacteria (injection challenge) than of those that received control injections (challenge control) (GLMM: df = 1, *X*^2^ = 23, *p* < 0.001; Figure 2B). Furthermore, offspring, whose grandfathers had received injection priming with heat-killed bacteria survived significantly better than those from the priming control group (GLMM: df = 2, *X*^2^ = 7.3, *p* < 0.05; THD: *z* = -2.492, *p* < 0.05; further comparisons: injection priming vs. naïve: *z* = -2.090, *p* = 0.09; priming control vs. naïve: *z* = -0.827, *p* = 0.68; Figure 2B). Therefore, the previously described TGIP in *T. castaneum* is transmitted past the first offspring generation at a comparable strength to the F_2_ generation.

**FIGURE 3:**
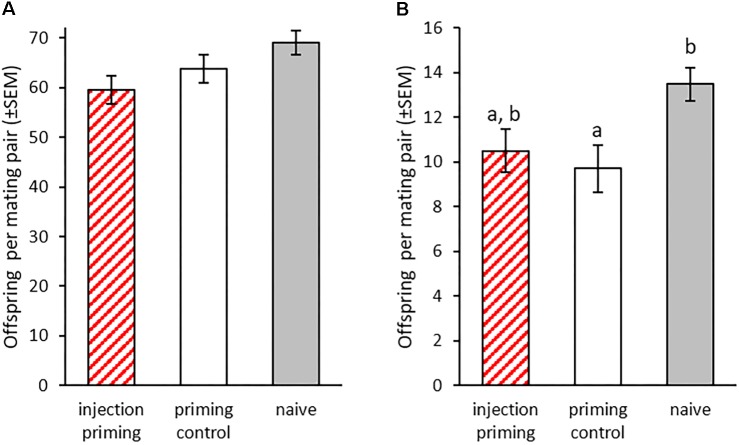
Fertility after injection priming with *B. thuringiensis* in adult males. **(A)** Mean offspring produced by injection-primed males within 6 days in single pair matings (*n* = 39–57) and **(B)** mean offspring produced by the offspring of injection-primed males within 48 h in single pair matings (*n* = 29–53). Different letters indicate significant differences at *p* < 0.05.

We investigated possible costs of paternal TGIP by counting live offspring 2 weeks after mating as a measure of reproductive success in the P_0_ and F_1_ generations. We could not observe any effect of paternal priming treatment on fertility for the P_0_ (GLM: df = 2, *X*^2^ = 3.399, *p* = 0.18; Figure 3A) nor the F_1_ generation (GLM: df = 2, *X*^2^ = 7.19, *p* < 0.05; THD: priming control *z* = -0.527, *p* = 0.86; naïve *z* = 2.014, *p* = 0.11, Figure 3B). However, the paternal priming control treatment significantly reduced fertility in the F_1_ generation and led to significantly less F_2_ larvae compared to the naïve control (THD: *z* = -2.381, *p* < 0.05; Figure 3B). Therefore, paternal septic wounding, but not the paternal bacterial priming itself, reduces the fitness of the F_1_ generation.

### Transgenerational Effects of Priming in Larvae

*T. castaneum* larvae can be either primed orally by feeding on filtered spore culture supernatant or through the direct introduction of heat-killed bacteria into the hemolymph by pricking or injection (Milutinović et al., 2014; Ferro et al., 2017). Both of these larval routes of priming have so far only been investigated within the same generation. We therefore here investigated whether any protection is transferred to larvae of the F_1_ generation. We further asked whether there are any costs associated with such larval priming.

#### Larval Priming Does Not Affect Fertility

Neither oral nor injection priming of larvae with spore supernatant or heat-killed bacteria, respectively, significantly affected fertility compared to the control groups or the naïve individuals (GLM: df = 4, *X*^2^ = 2.11, *p* = 0.71, Supplementary Figure S1).

#### Oral Priming Affects Development Differently in Treated (P_0_) and Offspring (F_1_) Generation

**FIGURE 4:**
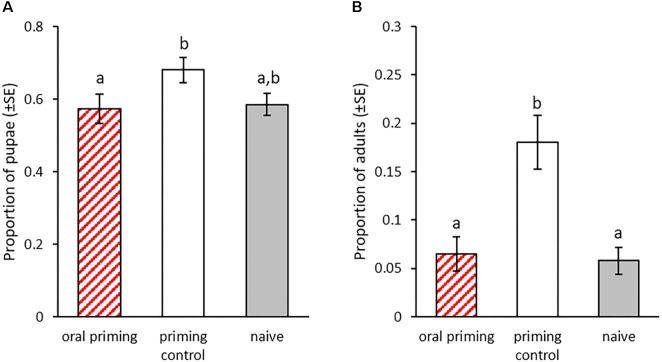
Development after oral priming during the larval stage. Priming with spore culture supernatant took place at 14 dpo for 24 h (*n* = 196–280). **(A)** Proportion of pupated individuals 23 dpo for nine replicates. **(B)** Proportion of eclosed adults 28 dpo for nine replicates. Different letters indicate significant differences at *p* < 0.05.

We monitored larval development after oral priming at 14 dpo to discover potential additional costs and benefits of this treatment besides changes in survival rate upon infection. In the treated P_0_ generation, there were significant differences in the pupation rates 21 to 25 dpo (Figure 4A). Larvae treated with *Bt407* supernatant (priming control), a bacterial strain that has been shown to not cause any immune priming (Milutinović et al., 2014) reached pupation faster than the *Btt* primed group (oral priming) (*z* = -2.906, *p* = 0.0102). There was also a trend toward earlier pupation of the priming control larvae compared to the naïve control (*z* = -2.28, *p* = 0.059), while the orally primed group and naïve control did not differ (*z* = -0.875, *p* = 0.65). Additionally, there were differences in time until adult eclosion (GLMM: df = 2, *X*^2^ = 17.52, *p* < 0.001; Figure 4B). At 28 dpo significantly more pupae from the priming control had eclosed than from the orally primed group (*z* = 2.98, *p* = 0.008) and the naïve control (*z* = 3.802, *p* < 0.001). Again, there was no difference between the orally primed and naïve control (*z* = 0.569, *p* = 0.84).

**FIGURE 5:**
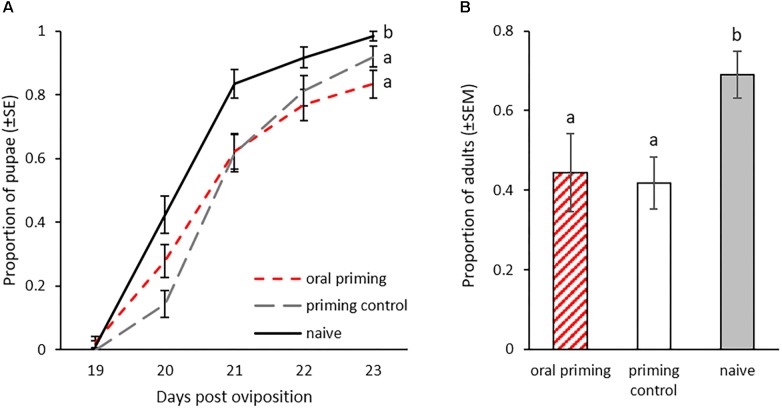
Offspring development after parental oral priming during the larval stage. Priming with spore culture supernatant took place in the P_0_ generation at 14 dpo for 24 h. Mating pairs were formed within the treatment groups. F_1_ generation larvae were individualized 14 dpo (*n* = 70–75). **(A)** Pupation rate of F_1_ generation (19–23 dpo). **(B)** Proportion of eclosed adults 28 dpo in the F_1_ for three replicates. Different letters indicate significant differences at *p* < 0.05.

We also observed the development in the F_1_ generation to see if this was influenced by the parental oral priming. Larvae, whose parents were exposed to spore culture supernatant from *Btt* or *Bt407* (oral priming and priming control) developed significantly slower than offspring of the naïve control (GLMM: df = 2, *X*^2^ = 16.14, *p* < 0.001; *Bt407*: *z* = 3.83, *p* = 0.002; *Btt*: *z* = 3.832, *p* < 0.001, Figure 5A). We found a similar effect for the development until adult eclosion, which on average was reached earliest by the naïve group (GLMM: df = 2, *X*^2^ = 14.17, *p* < 0.001; *Bt407*: *z* = -3.213, *p* = 0.004; *Btt*: *z* = -3.199, *p* = 0.004; Figure 5B).

#### No Survival Benefits of Oral Priming for the F_1_ Generation

To test whether the exposure to spore supernatants led to a trans-generational priming effect, i.e., increased offspring survival upon oral challenge, larvae of the primed P_0_ and the F_1_ generation were orally exposed to spores. In the primed P_0_ generation, the challenge with *Btt* spores (oral challenge) killed the larvae at a significantly higher rate than the exposure to spores of *Bt407* (challenge control) (df = 1, *X*^2^ = 12.76, *p* < 0.001; Supplementary Figure S2). This, however was regardless of priming treatment, which did not lead to any significant differences (df = 2, *X*^2^ = 0.63, *p* = 0.73; Supplementary Figure S2). This might be attributed to the here overall relatively low mortality rate after challenge with only 10.8% of all exposed larvae dying. This probably was caused by the rearing of larvae in lose flour instead of flour disks for the period between priming and challenge, because of which many larvae might have already had reached a pre-pupal stage and stopped feeding.

Although mortality was higher, results for the offspring generation were similar (Supplementary Figure S3). Again, the oral challenge proved to cause significant mortality at high (df = 1, *X*^2^ = 96.63, *p* < 0.001) and low concentration of spores (df = 1, *X*^2^ = 47.1, *p* < 0.001). Furthermore, survival depended on *Btt* spore concentration as the higher dose led to significantly higher mortality (df = 1, *X*^2^ = 10.85, *p* < 0.001). However, no effect of parental oral priming was observed (df = 2, *X*^2^ = 0.69, *p* = 0.71; Supplementary Figure S3).

#### Transgenerational Effects of Injection Priming in Larvae

**FIGURE 6:**
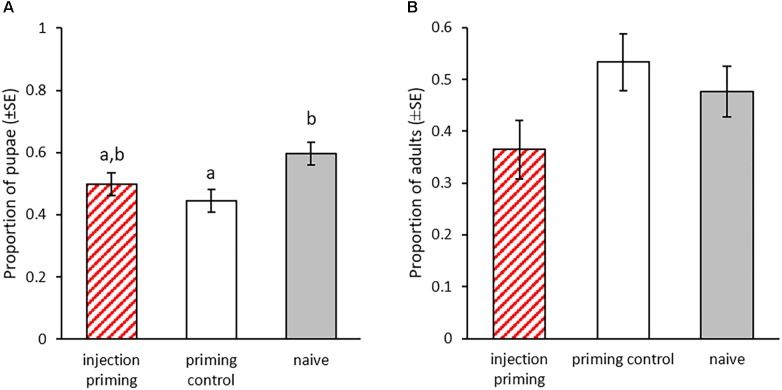
Development after parental injection priming during the larval stage. Priming with injection of heat-killed bacteria took place in the P_0_ generation 15 dpo. F1 was produced from mating pairs within the treatment groups. **(A)** Proportion of pupae for the P_0_ generation 23 dpo, i.e., 8 days post injection-priming (*n* = 196). **(B)** Proportion of eclosed adults for the F_1_ generation (parental injection-priming) 27 dpo for two experimental blocks (*n* = 72–103). Different letters indicate significant differences at *p* < 0.05.

In this part of the experiment we investigated potential effects of priming of larvae by injection with heat-killed bacteria. We monitored the development of the larvae after injection priming and the development of their offspring. Nine days after the priming, significantly less individuals from the priming control had pupated compared to the naïve control (X^2^ = 8.466, *p* = 0.003, Figure 6A). The addition of heat-killed bacteria to the injection reduced this effect, resulting in only a trend toward later pupation in the injection priming treatment compared to the naïve control (X^2^ = 3.74, *p* = 0.053, Figure 6A). There was no significant difference in the pupation rate between the injection primed individuals and the priming control (X^2^ = 1, *p* = 0.317, Figure 6A). In the F_1_ generation we did not observe any effect of parental priming on the developmental speed, as the eclosion rate was similar for all treatment groups at 27 dpo (GLMM: df = 2, X^2^ = 4.62, *p* = 0.1, Figure 6B).

We challenged the parental and offspring generation by injecting a potentially lethal dose of *Bt* at 19 dpo, i.e., 5 days after the priming procedures for the parental generation, when all individuals were still in the larval stage. As the majority of mortality occurred within 24 h of the bacterial injection, we did not use survival curves in the analysis, but instead used the survival rate differences after 7 days for our analysis.

In the P_0_ generation priming did not lead to differential survival after the injection challenge, which caused between 23 and 27% mortality (df = 2, *X*^2^ = 0.291, *p* = 0.86). Finally, in the larvae of the F_1_ generation, the bacterial injection challenge caused significantly higher mortality than the challenge control (GLMM: df = 1, *X*^2^ = 244, *p* < 0.001, Supplementary Figure S4). However, also in this case parental priming did not significantly increase survival as there were no significant differences in mortality rates between the parentally primed group and the two controls (GLMM: df = 2, *X*^2^ = 0.037, *p* = 0.98, Supplementary Figure S4).

## Discussion

*Tribolium castaneum* is one of the rare species for which not only maternal but also paternal TGIP has been observed (Roth et al., 2010, 2018). It is therefore important to further study this phenomenon. One of the major open questions regarding paternal TGIP is, whether it is effective in more than one subsequent generation and can be considered to be multigenerational. Additionally, it is important to understand what the costs of TGIP are and if these are also transferred to later generations. We therefore carried out bacterial priming and challenge experiments across three generations using adult beetles.

We found that offspring of primed grandfathers survived a bacterial challenge significantly better than offspring of grandfathers, which had received a priming control injection. Thus, paternal TGIP is persistent for multiple generations at least until the F_2_ generation. Astonishingly, the survival advantage of the F_2_ generation was at a similar level as observed in previous experiments for the direct offspring (Eggert et al., 2014). We therefore did not see any dilution effect of this phenomenon over subsequent generations. Furthermore, we witnessed indirect costs, not of TGIP itself, but of the wounding procedure during the injection. These fitness costs became only visible after two generations, when the offspring of fathers from the injection control group sired significantly less offspring. In the present experiment, in contrast to previous studies (Roth et al., 2010; Eggert et al., 2014), we were unable to detect a significant priming effect in the adult F_1_ offspring after paternal priming. This was likely due to an unusually high mortality in the injection control, maybe caused by a bacterial contamination in the injection buffer that was used for all treatments, thereby reducing a potential effect of priming.

Few studies have investigated the effects of TGIP beyond the first offspring generation. It has been shown that viral silencing agents derived from an RNAi response can be inherited non-genetically from either parent and passed on for several generations (Rechavi et al., 2011). In parthenogenetic *Artemia*, maternal exposure to bacteria provided the offspring with a survival benefit of bacterial infection for all three tested offspring generations (Norouzitallab et al., 2015). Multigenerational effects of paternal TGIP have been described in the pipefish, where due to male pregnancy contact between father and offspring is much more pronounced than in our system (Beemelmanns and Roth, 2017). Although, we are as of today unaware of the mechanisms behind paternal TGIP against bacteria, we can assume that its multigenerational nature will strongly impact the evolution of resistance and tolerance, depending on the costs, benefits, and specificity of TGIP and the prevalence of and therefore chances of repeated exposure to a parasite.

In the second part of this study, we investigated the transgenerational impact of immune priming via two different infection routes in larvae, for which within life stage immune priming has been previously demonstrated (Roth et al., 2009; Milutinović et al., 2014). Additionally to the survival after bacterial challenge, we monitored fitness costs of larval priming, becoming apparent as either directly reduced fertility or by slowing down developmental speed of the treated individual or its offspring. As any form of immunity, also immune priming comes at a cost for the organism (Schmid-Hempel, 2005; Freitak et al., 2009; Sadd and Schmid-Hempel, 2009). While in mosquitos a trade-off between immune priming and egg production has been observed (Contreras-Garduño et al., 2014), we did not find any effects of priming on fertility. Similar numbers of live offspring were produced across all treatments for both priming methods. But we estimated fertility only from a short 48h reproduction period and do not know how the immune priming might affect lifetime reproductive success. Also, we provided the beetle with *ad libitum* food throughout the experiment, whereas limiting resources can be necessary for uncovering trade-offs with immunity (Moret and Schmid-Hempel, 2000; Kutzer and Armitage, 2016).

However, the oral priming of larvae led to differential speed in their development. Larvae, which had received the priming control diet containing the supernatant from the *Bt407* culture reached pupation considerably faster and emerged as adults earlier. In contrast, the treatment with *Btt* did not lead to differential developmental time compared to the naïve larvae. The same effect was observed previously by Milutinović et al. (2014). It is possible that the supernatant from the *Bt407* control culture contained some nutrients that were transferred to the priming diet and helped the larvae to speed up their development. The supernatant from the treatment *Btt* culture might not contain these nutrients, due to differences in the bacteria. Alternatively, the necessity to mount an immune and priming response, brought on by the exposure to the priming diet might mitigate the potential effect of the additional nutrients.

In the offspring generation, development was strongly affected by parental larval treatment. Both, offspring from the *Btt* primed group and the *Bt407* priming control took longer to pupate and also emerge as adults. This is interesting because although *Bt407* does neither provide an immune priming (Milutinović et al., 2014) nor is able to kill larvae upon ingestion (Milutinović et al., 2013), larvae feeding on its spore supernatant still suffer these fitness costs. These results are in concordance with observations in the mealworm beetle, where maternal priming prolonged larval development, while paternal priming led to a reduction in larval body mass (Zanchi et al., 2011). For the injection priming, we only observed within generation effects on the development. Here the wounding by the injection was sufficient to cause the effect, because larval development was slowed down in the injection of heat-killed bacteria as well as in the injection control treatment compared to the naïve group. Similar delays in development and increased time until pupation after tissue damage were observed in *D. melanogaster* (Halme et al., 2010). In the fly and potentially also the beetle, tissue damage interferes with endocrine signals, which are essential for the progression of development (Halme et al., 2010). In the offspring generation, time until adult emergence was not affected by parental priming. So far, we have no data regarding the development until pupation in this case.

Increased developmental time during the larval and pupal stage can be considered a fitness cost. Longer time spend during the larval stage is costly as it increases several risks. During the larval stage the risk of infection is higher as only larvae can be infected orally with certain bacteria, including *Btt*. Also, there is a higher risk of cannibalism, which happens regularly among larva (Ichikawa and Kurauchi, 2009) and at high densities smaller larvae might be less able to secure sufficient food (Koella and Boëte, 2002). Therefore, prolonged development should decrease probability of survival and delay the start of reproduction. In this experiment we were unable to confirm within-generation immune priming for either of the two used infection methods. This can likely be attributed to the low overall mortality rates following the challenge, which is a problem occasionally encountered in such experiments (see also Tate et al., 2017). However, both within-generation priming methods have been shown to work consistently in our lab (Milutinović et al., 2014; Ferro et al., 2017; Futo et al., 2017).

We did not find any evidence of larval TGIP with the oral nor the injection protocol. For larval priming by septic wounding with a pricking needle, it was observed that TGIP in larvae only occurred in populations, which do not demonstrate within life stage immune priming (Khan et al., 2016), implying that they are incapable of developing and maintaining both forms of immune protection. As beetles from our population have repeatedly been shown to possess larval within life stage priming ability, this is a possible explanation for the absence of larval TGIP.

In the present study, we did not directly address the question of potential mechanisms underlying immune priming. Nevertheless, our results indicate that such mechanisms should enable reactions that can be transferred not only within the organism (i.e., systemic reactions) but even across generations up to the F_2_. This might be helpful for narrowing down targets for further in-depth studies from the large range of candidate genes and mechanisms identified in *T. castaneum* (Ferro et al., 2017; Greenwood et al., 2017; Tate et al., 2017; Schulz et al., 2018) and other insect species (e.g., Castro-Vargas et al., 2017; Tassetto et al., 2017; Cime-Castillo et al., 2018; Mondotte et al., 2018). On a more cautionary note, our study also further supports the view that immune priming comprises a multitude of phenomena that might be based on diverse mechanisms among and even within species (for review see Contreras-Garduño et al., 2016; Milutinović and Kurtz, 2016).

In conclusion, we observed that the life stage and route of priming determine the effects on the next generation. We found that the parental priming can be transferred to the F_2_ generation, but can also impact offspring development. This demonstrates long-term costs of immune priming that are paid by subsequent generations. As this might have fitness consequences, further experimental research might focus on the evolution of immune priming. This will help to clarify under which circumstances immune priming is favored over the evolution of resistance or tolerance (Tidbury et al., 2012; Tate, 2017). Such knowledge will also be helpful to understand the evolutionary consequences of pest control methods that make use of bacterial products such as toxins derived from *B. thuringiensis*, as these may lead to priming in natural populations of pest organisms.

## Data Availability

The raw data generated for this study can be found in the Supplementary Material (Supplementary Data Sheet).

## Author Contributions

NS, MS, KF, and NK performed the experimental work. NS performed the statistical analysis and drafted the manuscript. All authors contributed to conception, design of the study, manuscript revision, and read and approved the submitted version.

## Conflict of Interest Statement

The authors declare that the research was conducted in the absence of any commercial or financial relationships that could be construed as a potential conflict of interest.
