# Supplementary material for: Transgenerational Developmental Effects of Immune Priming in the Red Flour Beetle Tribolium castaneum
Source: Front Physiol. 2019 Feb 19;10:98. doi: 10.3389/fphys.2019.00098 (PMC6389831; doi:10.3389/fphys.2019.00098)
Supplement: Supplementary file 2 [file Image_1.pdf]

Transgenerational developmental effects of immune priming in the red flour beetle Tribolium castaneum

Nora KE Schulz[1], M Pauline Sell[1], Kevin Ferro[1], Nico Kleinhölting[1], Joachim Kurtz[1*]

[1]Institute for Evolution and Biodiversity, University of Münster, Münster, Germany

*** Correspondence:**
Joachim Kurtz
Joachim.kurtz@uni-muenster.de

Supplemetary information

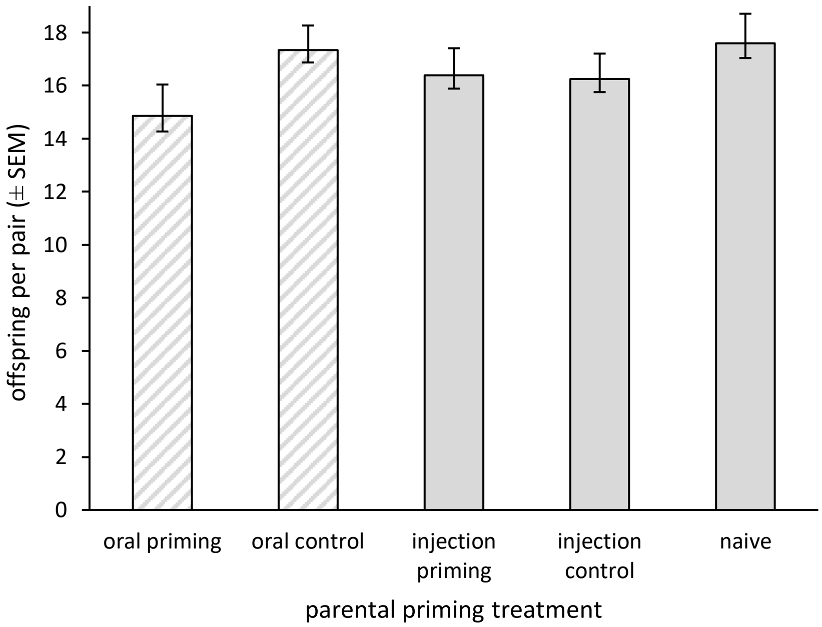

Figure S1 Fertility after oral and injection priming of larvae. Total live offspring from two consecutive 24 h ovipositions (n=57-66 mating pairs). Oral priming was performed with filtered supernatant from *Btt* (oral priming) or *Bt407* (oral control) spore culture. Injection priming was performed with heat-killed vegetative *Bt* cells. Injection control consisted of PBS buffer and a naïve group served as handling control. Live larval offspring was counted 14 dpo.

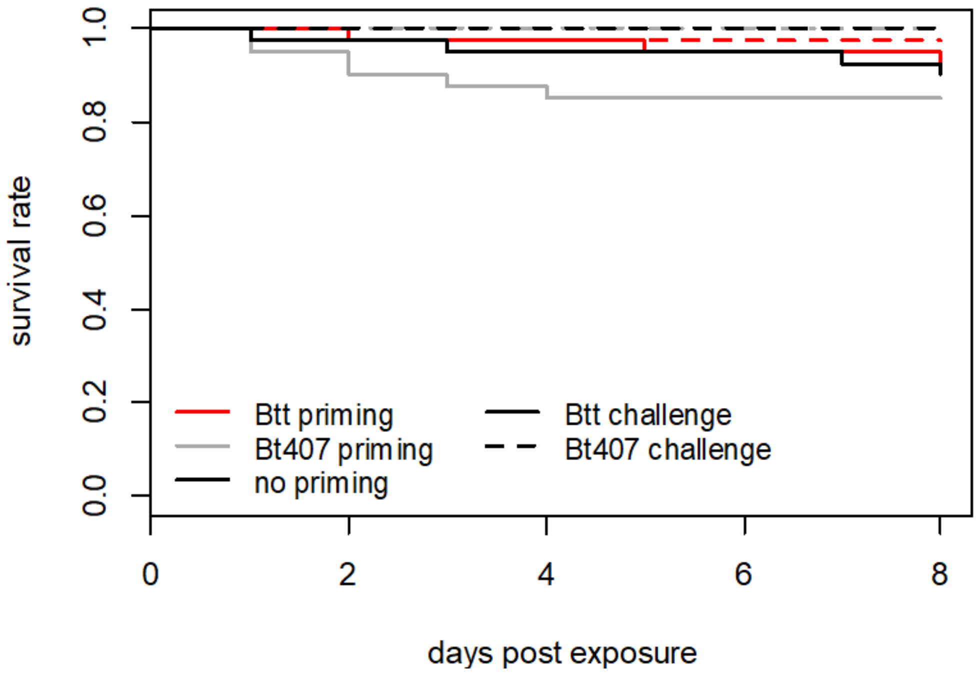

Figure S2 Survival after oral challenge after larval priming within generation (n=40). Five days after larval oral priming with spore culture supernatant (oral priming = *Btt*, priming control = *Bt407*, naïve =no priming) larvae were exposed to potentially lethal diet of *Btt* spores or a control diet of *Bt407* spores.

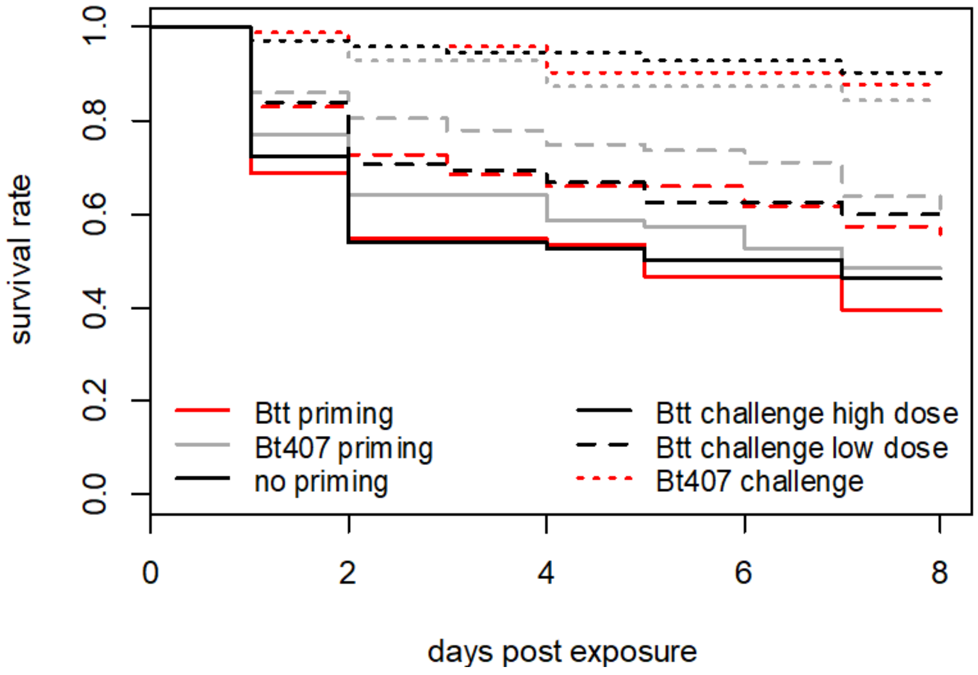

Figure S3 Survival after oral challenge of the offspring generation after parental oral priming (n=71-76). Two concentrations of *Btt* spores (low=5*10^9, high=1*10^10) and *Bt407* spores were used to challenge 19 days old larvae of the F_1 generation.

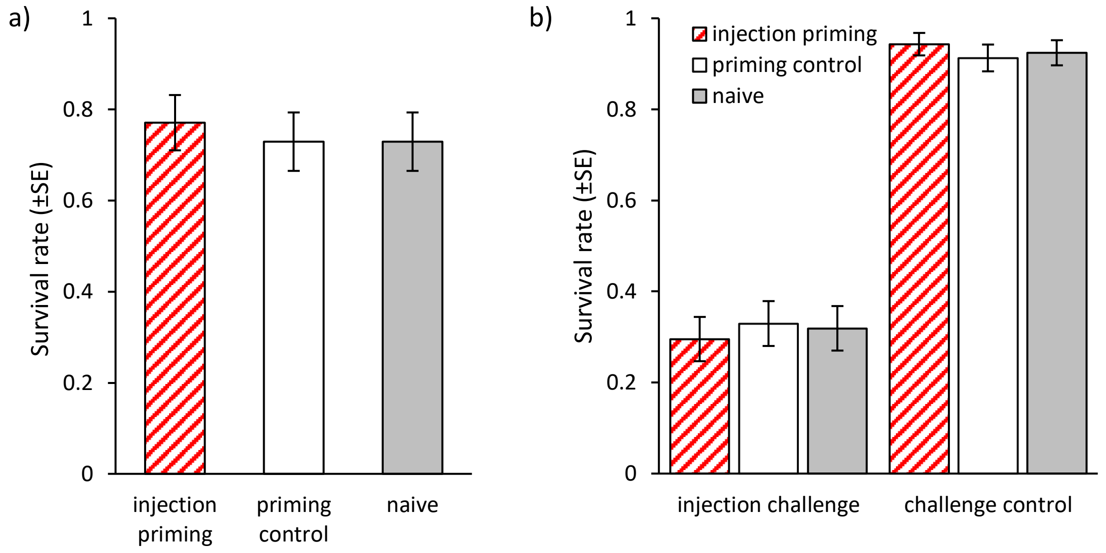

Figure S4 Survival after injection challenge a) Larvae from the parental generation were injected with live *Bt* cells(n=48) and their survival was recorded 24 h later. b) Larvae of the F_1 generation after parental injection priming were either injected with live *Bt* cells or PBS buffer as a control (n=96). Survival was recorded 24 h after treatment.
